# Contemporary mass balance on a cold Eastern Alpine ice cap as a potential link to the Holocene climate

**DOI:** 10.1038/s41598-021-04699-2

**Published:** 2022-01-25

**Authors:** Andrea Fischer, Martin Stocker-Waldhuber, Martin Frey, Pascal Bohleber

**Affiliations:** 1 Institute for Interdisciplinary Mountain Research, Austrian Academy of Sciences, Innsbruck, Austria; 2 Department for Environmental Science, Informatics and Statistics, Ca’Foscari University of Venice, Venice, Italy; 3 Poschenhof, Kaunertal, Austria

**Keywords:** Cryospheric science, Palaeoclimate

## Abstract

Alpine cold ice caps are sensitive indicators of local climate. The adequate interpretation of this information in an ice core requires detailed in situ glaciological and meteorological records, of which there are few. The Weißseespitze summit ice cap (3499 m) presents an ideal case to compare past and present climate and mass balance, with limited ice flow, but close to 6000 years locked into about 10 m of ice. First-ever meteorological observations at the ice dome have revealed that over 3 years of observation most of the accumulation took place between October and December and from April to June. In the colder winter months, between January and March, wind erosion prevents accumulation. Melt occurred between June and September, ice was only affected during short periods, mainly in August, which caused ice losses of up to 0.6 m (i.e. ~ 5% of the total ice thickness). Historical data points at a loss of of 34.9 ± 10.0 m between 1893 and 2018 and almost balanced conditions between 1893 and 1914. The local evidence of ice loss lays the basis for the interpretation of past gaps in the ice core records as past warm/melt events.

## Introduction

Glaciers are icons and indicators of climate change and mitigation efforts^1^. The current Central European glacier retreat is historically unprecedented^2^ and results in large-scale down-wasting^3^. Therefore, records of past and present glacier state and fate are important, not only for science but also for society. Current monitoring is designed to cope with the role of glaciers as essential indicators of past climate conditions^4^. In the European Alps, records of glacier variability go far back^5^ and thus offer the most direct link between instrumental data and glacier area or mass balance as proxy records of climate. Climate information from Alpine glaciers includes past terminus positions recorded in the positions of moraines deposited during past glacier advances^6^, the structure and age of glacial deposits^7^, measured length changes as well as total and point mass balances. The timing and extent of glacier advances is greatly influenced by topography and dynamics^8^, and so are proxy data of terminus positions.

The role of glaciers as climate archives is unique. It ranges from establishing the idea of a changing climate^9^ to polar ice cores as cornerstone archives of past climate and atmospheric composition^10^. With the deposition of snow, additional properties of the atmospheric composition are archived (aerosols, stable water isotopes, etc.). In the absence of prolonged mass loss, a chemical and structural stratification of ice exists which makes up the paleoclimatic record that can be accessed by drilling ice cores. However, ice core records from the polar regions are not necessarily representative for middle and low latitudes, especially for short-lived atmospheric constituents.

At least for the Holocene period, important complementary information can come from ice cores drilled at cold (non-temperate) glaciers and ice caps of high mountain ranges in nonpolar areas^11^. In the European Alps, ice core research aimed at continuous stratigraphic climate records focused on a few suitable drilling sites in the Western Alps. There, in locations above 4000 m, the low infiltration depth of (refreezing) meltwater, if it occurs at all, allows the preservation of the original layering of the accumulation^12^.

Recent studies have shown that also in the Eastern Alps, and at elevations below 4000 m, millennial-old ice and associated climate records may be preserved^13–15^, sparking new efforts to retrieve this valuable paleoclimatic information which is currently threatened by warming^16,17^. In view of the predominant mass loss even at the summits of such sites, it is paramount to also take into account the role of surface mass balance in the interpretation of the associated ice core records. This opens up a new field of research questions, linking mass and energy balance consideration and modelling efforts to aid the interpretation of ice core records^17^.

Direct mass balance measurements on summit glaciers complement paleo-mass balances derived from ice cores^18^, supported by numerical modelling^17^. As the age-depth profile of ice cores is not only relevant for paleo-hydrology and glaciology, but also for the interpretation of ice core records, this becomes a field of major significance.

Geodetic or direct mass balances of glaciers include a feedback from glacier geometry. Several authors have presented methods to estimate the influence of those geometrical feedbacks, such as^19,20^. Strategies employing reference surfaces^21,22^ and point mass balances^23^ aim to discount the topographical and dynamical components.

**Figure 1:**
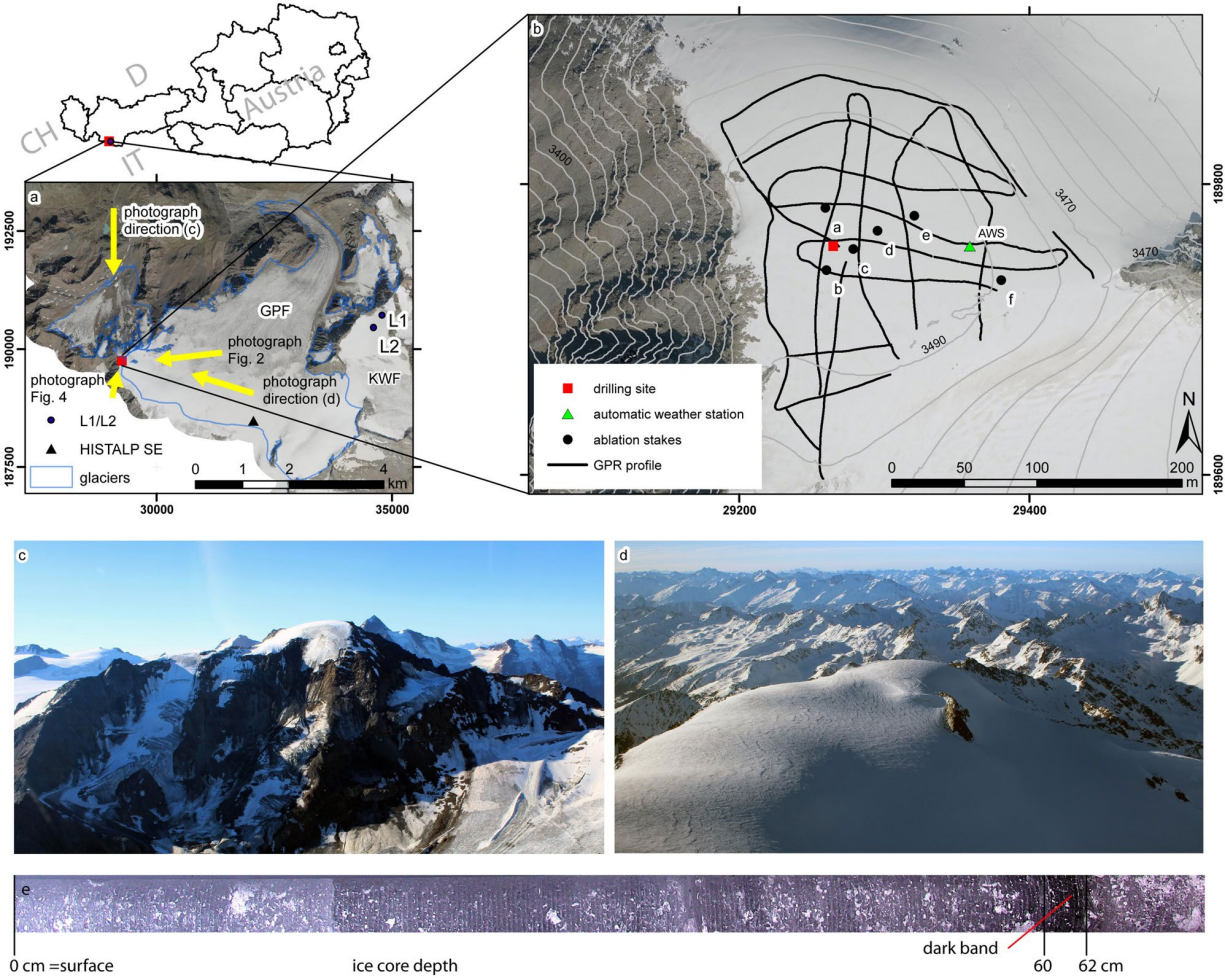
(**a**) The drilling site at the summit glacier at Weißseespitze (red square) is part of Gepatschferner (GPF); on neighbouring Kesselwandferner glacier Austria’s previously highest altitude stakes have been operated since the late 1960s; (**b**) ice thickness has been measured with GPR, ablation stakes were drilled (**a–f**), an automatic weather station (AWS) was installed and an ice core was drilled in March 2018 (15). Photos from the north (**c**) and east (**d**) show the summit ice cap at Weißseespitze (viewing directions: yellow arrows in (**a**). The visual ice core stratigraphy (**e**) shows a dark layer and bandings of variable inclination and bubble density. The relation of these features with regards to an atmospheric or glaciological origin remains unclear. Only a detailed understanding of the physical processes governing the mass balance can fill this gap. This figure was created with Adobe Illustrator CS4 version 14.0.0 (www.adobe.com). The maps were compiled with ArcMap 10.6.1 (www.arcgis.com).

The logical consequence of such attempts is studying point mass balances at cold summit glaciers, which are frozen to the ground and thus almost stagnant. So far, only very few summit stakes have been installed within traditional and long-term mass balance programs. During the 1960s to 1980s, when most of today’s benchmark glacier in situ programs started, it took immense efforts to dig out and replace the stakes, so numbers were kept to a minimum. These point mass balance data sets, often unpublished and difficult to find, have already been compiled for the Swiss Alps^24^. For the Austrian Alps, single high elevation stakes have been in operation, e.g. at Kesselwandferner glacier (3494 m, Fig. 1^25^) from 1968 to 1983 and at Übergossene Alm (2850 m) from 1965 to 1975^26^. Thickness records at least are available from repeat cartographic campaigns, where the summit elevation was often measured directly, e.g. for the Weißseespitze summit (3531 m anno 1922) at Gepatschferner glacier^27^.

In the Western Alps, several prominent glaciers in summit locations have been investigated in detail, for example in the Monte Rosa and Mont Blanc region^28–30^. Although high-elevation glaciers, especially in the Western Alps, were remarkably untouched by global warming^31^, englacial warming has been recorded despite insignificant volume changes^32^. Glacier outlet floods of meltwater trapped by still cold glacier parts also threaten, as happened at the Tête Rousse glacier cavity^33^.

The present paper analyses present mass balance and more than a century of thickness change evidence as a basis for the detailed interpretation of the stratigraphy of the ice core drilled at Weißseespitze (3499 m, Fig. 1). The ‘present’ direct mass balance and thickness changes were recorded during 3 years at subseasonal resolution and set a baseline for the last ~ 125 years of ice elevation changes from geodetic data. This first presentation of mass loss data is compared in detail to the meteorological conditions measured at the summit, published previously as an overview, and to the total thickness of the remaining ice cap comprising nearly 6000 years of ice 15.

Constraining the contemporary annual and decadal mass balance and its relation to instrumental climate data is a necessary and important step to link the ice core stratigraphy potentially representing past mass balances and past climate. This scientific effort is important to identify how and in what way current processes are potentially unprecedented. This study contributes to our understanding of present glaciological processes on summits, the respective meteorological conditions and their variability to improve the interface to past mass balance archived in summit ice cores.

## Results

### Meteorological conditions at the summit 2018–2020

**Figure 2:**
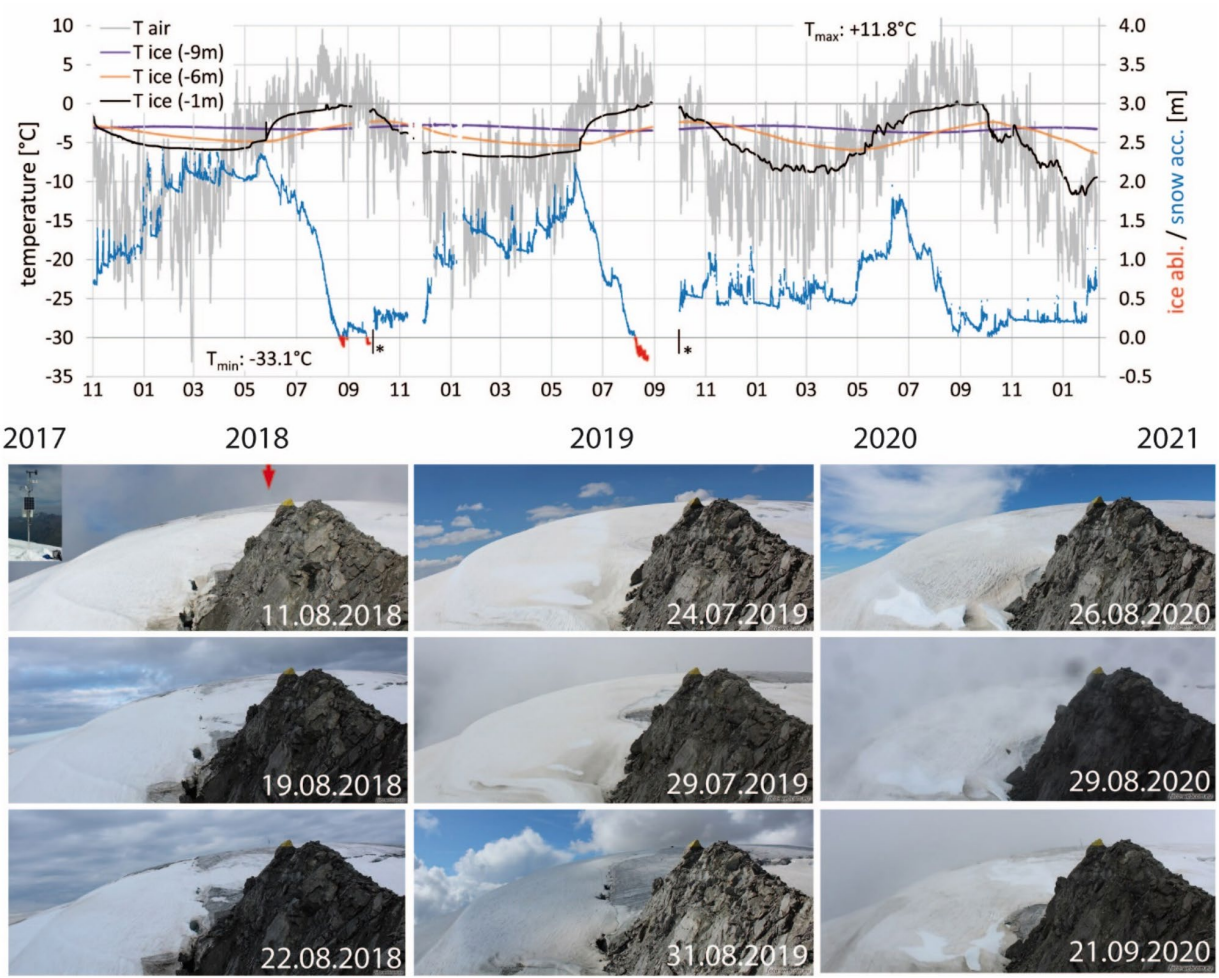
Air temperature (T air) and ice temperatures (T ice) at different depths related to the 2017 ice surface, from November 2017 to February 2021, as well as the minimum and maximum temperature for the whole period. Snow accumulation and ice ablation measured with the sonic ranging sensor refer to the right ordinate axis. Ice ablation (red lines) took place from 22.08.2018 to 27.08.2018 and 09.08.2019 to 31.08.2019 2019 (end of period reconstructed from camera images) (*). In 2020, no ice melted at the location of the AWS. New distance to surface correction of the sonic ranging sensor sets the ice surface to zero. The pattern of mass balance is visible on the time-lapse camera images (positon of the AWS indicated by the red arrow).

The meteorological conditions at the summit were recorded at the automatic weather station (AWS) between November 2017 and February 2021 (Fig. 2). The mean annual air temperature was − 7.6 °C in 2018 and − 6.1 °C in 2019 and 2020, with an absolute minimum of − 33.1 °C and a maximum of 11.8 °C. Only very limited increase in snow cover was recorded between 27.01.2018–11.04.2018, 17.12.2018–02.05.2019 and 22.10.2019–27.04.2020. During cold conditions, snow accumulation seems to be limited by wind erosion (Fig. S1). Snow height increases mainly between October and December and in June (Fig. 2). The three ice temperatures at different depths show a seasonal signal. Mean annual ice temperature at the lowest sensor is around − 3.3 °C, maximum values do not exceed − 2.6 °C (Table S1).

### In situ mass balance

Glacier mass balance measurements at Weißseespitze summit comprise automatic measurements of snow height and ice thickness loss in subdaily resolution at the AWS and stake measurements in subseasonal resolution. During the 3 years of observation, snow height maximum values appeared in May and June, with an all out maximum snow depth of 2.4 m recorded in May 2018. During winter 2019/2020, snow height was around 0.5 m until May and reached its maximum with 2.2 m in June. Winter 2020/2021 presents a similar pattern, with less than 0.5 m snow height until February.

Continuous records of ice ablation indicate the duration of the ablation season and the magnitude of ice melt at the AWS. Data gaps cause an underestimation of the seasonal ablation values measured, with 0.11 m in 2018 and 0.29 m in 2019. The length of the ablation season at the weather station was corrected by time-lapse camera images. No ablation was recorded at the AWS during summer 2020.

At the ablation stakes, the spatial pattern of ice ablation is highly variable between years depending on the weather conditions and snow accumulation patterns (Fig. 2), as evidenced by stakes c and f. There we recorded similar ice melt in 2017 and 2018, but not in 2019 (Table 1). The positions of the stakes were recorded several times a year with DGPS, together with fixed control points in the surrounding area. The stake positions did not change within the DGPS accuracy, proving the stagnancy of the ice dome at the summit above the bergschrund.

**Table 1:** Annual surface ablation measured at stakes a-f and at the drilling site [m ice] as well as the ice ablation and maximum snow height (max Sh) from the sonic ranging sensor at the automatic weather station (AWS).

	a	b	c	d	e	f	Drilling site	AWS	mean	max Sh in m
2017			0.54			0.76			0.65	
2018	0.95	0.82	0.81	0.61	0.28	0.83		0.11	0.63	2.43
2019	0.40	0.00	0.63	0.43	0.75	0.00	0.26	0.29	0.35	2.24
2020	0.00	0.23	0.00	0.00	0.08	0.00	0.00	0.00	0.04	1.80

The AWS values are generally too low due to data gaps towards the end of the ablation seasons (Fig. 2).

### Ice thickness distribution, subsurface topography and volume changes

**Figure 3:**
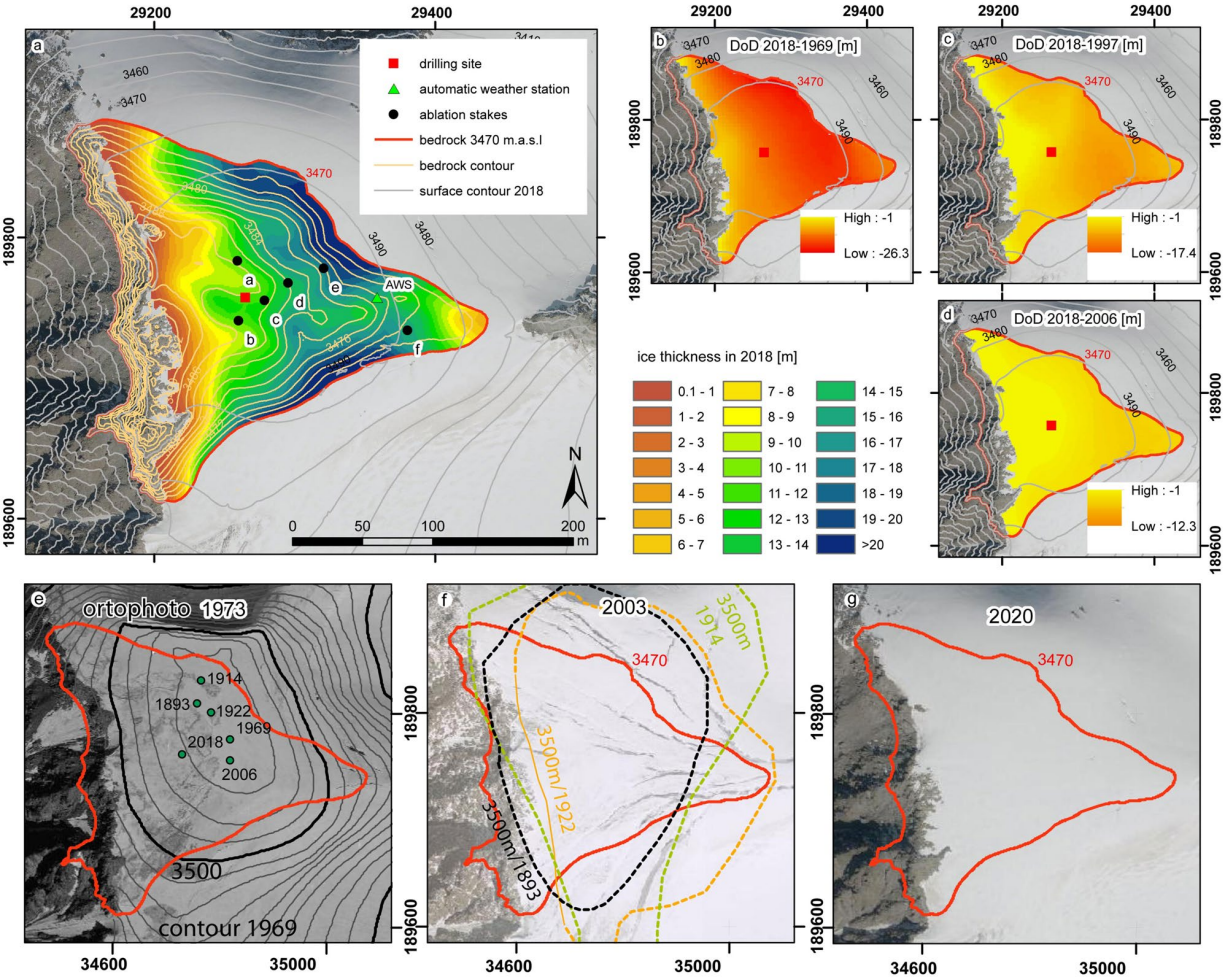
Bedrock topography from the GPR measurements and calculated ice thickness in 2018 (**a**). The region above the 3470 m bedrock contour was chosen as area of interest for comparing maps of thickness change (DEMs of Difference, DODs) for the periods 2018–1969 (**b**), 2018–1997 (**c**) and 2018–2006 (**d**). The position of the measured maximum altitude and the past contour lines of elevation shows that all of them are located within the 3470 m bedrock altitude band (**e**), as well as above the 3520 m contour line of 1969, and that past cartographers drew the 3500 m contour line in order to represent the dome shape evident from the photographs (**f**). The orthophotos of 1973 are the only available ones so far where signs of melt at the summit dome are evident, as (**e–g**) demonstrate. This figure was compiled with Adobe Illustrator CS4 version 14.0.0 (www.adobe.com). The maps were created with ArcMap 10.6.1 (www.arcgis.com).

The Weißseespitze summit forms a glacier dome with circular contour lines from about 3470–3500 m (Fig. 1). A bergschrund reveals the stagnant summit region (Fig. 3). From the top, the ice thickness increases sharply towards the north-eastern and the south-eastern part and shrinks to zero towards the ridge of the west face. In 2018 the mean ice thickness above the 3470 m bedrock contour was 12.1 m, when the maximum altitude of the glacier summit surface was 3499 m. The maximum elevation from DEMs in 1969 is given as 3518 m (Table 2). Maximum elevations from historical maps suggest an LIA maximum (1855 AD) elevation only slightly above 3534 m (Table S2). The position of the highest point at the summit has shifted within a distance of about 100 m (Fig. 3). Currently the highest point is located just a few metres from the drilling site.

**Table 2:** Maximum elevation (h_max_) of the summit Weißseespitze from DEMs and historical maps^27,34–37^, as well as the mean thickness change h_mean_ (Fig. 3, Table S2) in total and per year.

Year	h_max_ (m)	Period	Length (years)	Δh_max_ (m)	Δh_max_/a (m/a)	Δh_mean_/a (m/a)
2018	3499.1 ± 0.1	2018–2006	12	− 7.5	− 0.6 ± 0.1	− 0.2 ± 0.1
2006	3506.6 ± 0.1	2006–1997	9	− 0.4	0.0 ± 0.1	− 0.3 ± 0.1
1997	3507.0 ± 1.9	1997–1969	28	− 11.0	− 0.4 ± 0.2	− 0.3 ± 0.2
1969	3518.0 ± 1.9	1969–1922	47	− 13.0	− 0.3 ± 0.2	− 0.1 ± 0.4
1922	3531 ± 1.9	1922–1914	8	− 3.0	− 0.4 ± 1.3	− 1.0 ± 2.5
1914	3534 ± 10	1914–1893	21	0.0	0.0 ± 0.5	− 0.6 ± 1.2
1893	3534 ± 10					

The maps of thickness changes show greatest losses from the north-eastern to the south-eastern sector and smallest losses towards the west face (Fig. 3). The mean elevation changes for the area above the 3470 m bedrock contour line (Fig. 3) range from − 0.3 (2006–1997) to − 0.2 m (2018–2006) per year. From 1969 to 2018, the longest available period covered by modern DEMs, the mean elevation changed by − 15.5 m (Table S3). During this period, the summit area lost 56% of its ice volume. The total remaining ice volume in 2018 is 0.38 × 10^6^ m^3^ (Table S2).

In the historical maps, the maximum thickness change was directly measured. The spacing of the manually interpolated contour lines of altitude in the historical map varies between 10 and 50 m. This causes an uncertainty about the shape of the summit, which can be greater than the expected altitude change. Therefore, we used the directly measured maximum altitude, indicated by respective map symbols, as a proxy to estimate long-term changes in mean thickness (Table 2).

### Historical climate records as a baseline for modern in situ data

**Figure 4:**
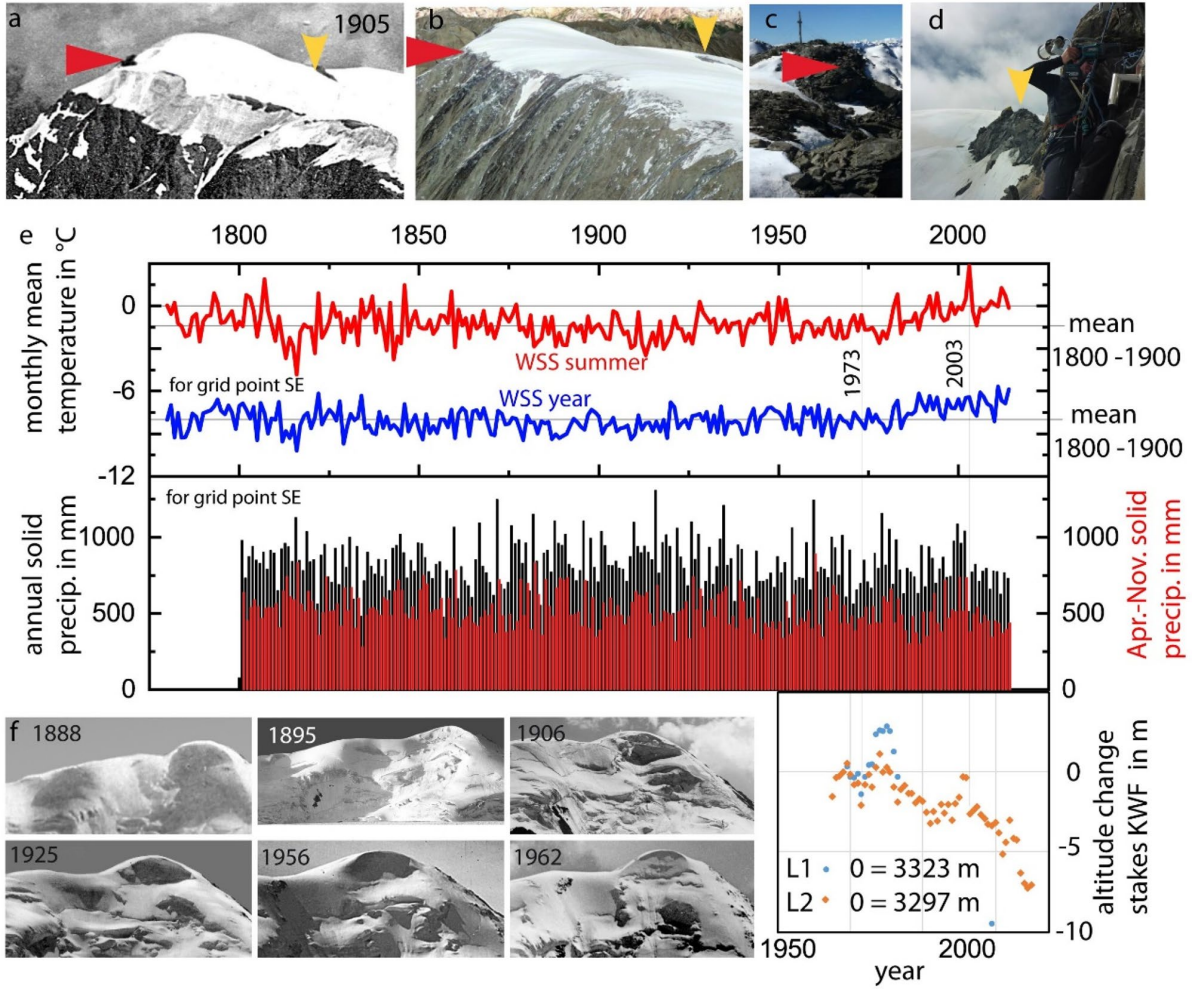
The comparison of the (**a**) historical photograph from 1905 with (**b**) one of 12.08.2021 supports the maximum elevation change of 34.9 ± 10.0 m between 1893 and 2018 and shows that the (**c,d**) rock ridges marked by arrows in (**a–d**) were ice-free during the late LIA. (**e**) Mean annual and summer temperatures for the HISTALP grid point SE extrapolated to Weißseespitze show an increase starting in the 1980s. Solid precipitation for the total year (black bars) and April–November (red bars) both decrease in the twenty-first century. The thickness at stakes L1 and L2 at Kesselwandferner increased with solid precipitation peaks in the 1980s and late 1990s, and decreased with melt events, e.g. in 1973 and most remarkably since 2003. The time series of historical photographs (**f**) of Weißseespitze summit shows varying steepness of the ice cliff, but an unchanged dome shape. Data Source: HISTALP temperature and precipitation data^40^, federal administration of Tyrol data.tirol.gv.at, photo by Andrea Fischer^27,34–37^.

Mean summer temperatures have been known as indicators of glacier melt for more than 100 years, even though on Alpine glaciers the main source of energy governing glacier melt stems from direct radiation^38^. Climate change impact on mass balance is described by changes in the sum of positive daily temperature means and precipitation^39^. For Weißseespitze, gridded Historical Instrumental climatological Surface Temperature and precipitation time series of the Greater Alpine Region (HISTALP) is available only as monthly means/sums for 1800–2014 (Fig. 4).

We compared the mass balance records to AWS data to find out if local ice melt is indicated by monthly mean temperatures. In a second step, the AWS records were compared to long-term HISTALP data to find if the monthly or seasonal means directly measured at WSS exceed the long-term average.

**Figure 5:**
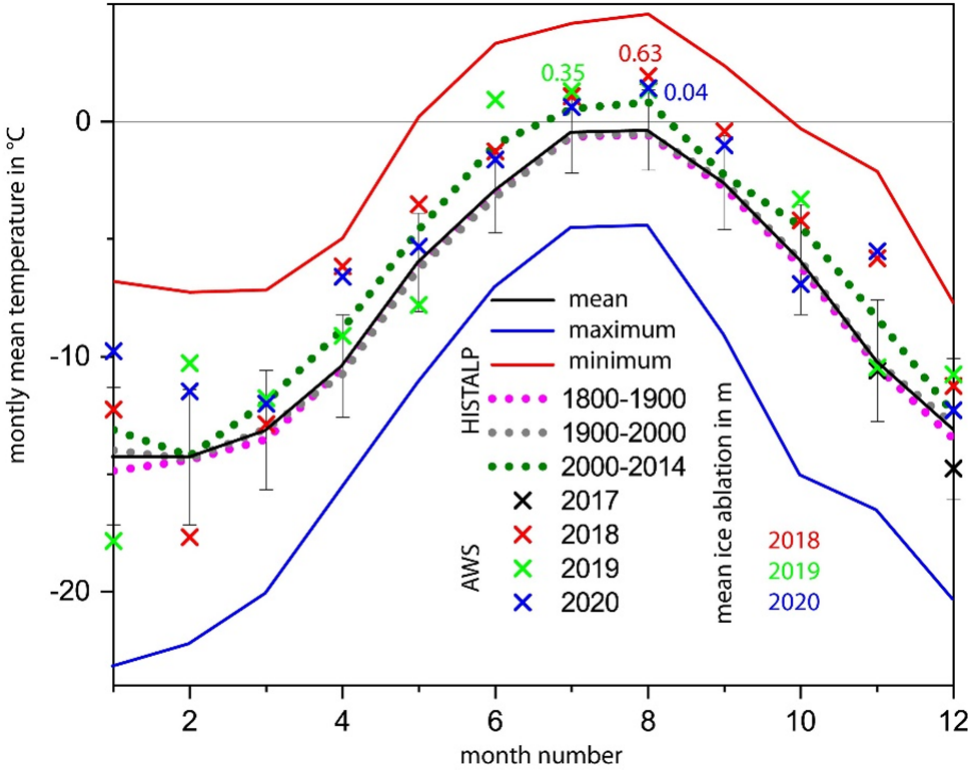
Mean, maximum and minimum monthly means for the total length of the record (1780–2014, Table S4) show that, on average, monthly means are below zero even in summer, but positive mean temperatures have occurred between May and October. The temperatures observed at the AWS are clearly above the mean, even for 2000–2014. Centennial averages are close to the mean and below zero.

In 2018, the decrease in snow cover started on 24.05., interrupted by several snow falls until 24.07.2018. Ice melt was recorded at Weißseespitze summit AWS during 22. and 27.08.2018 as well as between 24.09.2018 and 1.10.2018. Monthly air temperatures have been above the freezing point in July (1.1 °C) and August 2018 (1.9 °C) and close to 0 °C in September 2018 (− 0.4 °C), as well as in July 2019 (Fig. 5). In 2019, snow cover decrease started on 29.05. and was shortly interrupted by a snow fall on 29.05.2019. Ice ablation took place between 09.08 and 01.10, with monthly means of air temperature above 0 °C for June, July and August and a data gap in September. In 2020, snow cover decrease started on 29.05. and was interrupted by several snow falls until 30.08. when the ice surface was exposed. In contrast to some of the stakes, no ice melt took place at the AWS. Monthly mean averages in 2020 were positive in July and August. The limited sample of available data indicates that monthly means of local air temperature close to or above the freezing point coincide temporally with the observed ice melt.

In the HISTALP record, the number of months with positive mean air temperatures increased most pronouncedly around 1800 AD and after 1984 AD (Fig. 4). With the stability of the precipitation signal in the HISTALP record during the accumulation period for the study site, we can thus claim that mass balance at Weißseespitze summit ice cap is related to air temperature.

The variability of solid precipitation is high, but the solid precipitation during the months when accumulation takes place has decreased since 2002. May/June and October/November contribute most to solid precipitation (Table S5), about 10% of the mean annual amount each. During the cold months of January to April, when the ultrasonic sensor at the AWS records a plateau in snow height, usually no accumulation can take place as the snow is removed by wind erosion, even though the HISTALP record shows that 29% of the annual solid precipitation falls in this period.

## Discussion

The in situ and geodetic mass balance rates confirm that climate warming in the twentieth century significantly affected the ice archive at the summit of Weißseespitze. By 2018, the 12.1 m thick summit ice cap had lost 56% of its thickness of 1969. In the four years of direct mass balance measurements, the average annual ice loss was 0.4 m. The historical thickness loss from 1893 to 1969 at the highest point was 16 ± 5 m. Comparing the annual thickness loss of 0.2 m for 1893–1969 and 0.3 m for 1969–2018, we must take into account that the summit was covered by firn prior to the 2010s.

The present ablation measured at the stakes, with up to 0.95 m of ice loss, has removed more than the total annual accumulation in the past, although ablation conditions last just a few days. The measured mean mass balance rates would mean a total loss of the ice cap within 30 years, measured maximums a loss within 12 years. This means that the 12.1 m of ice in 2018, which comprise more than 5900 years, could not have continued to exist across this period under current melt rates. Current melt rates are thus (i) higher than the average of the last 6 millennia and (ii) past similar events must have led to gaps in the ice core stratigraphy. The visual stratigraphy of the Weißseespitze ice core already shows a number of layers that suggest discontinuities, such as alternating sections of inclined and horizontal layers and a visible dark band (Fig. 1). These features are currently under further investigation, including obtaining precise chronological constraints. Weißseespitze and similar summit ice caps can thus be considered very sensitive indicators of past warm summer conditions.

**Figure 6:**
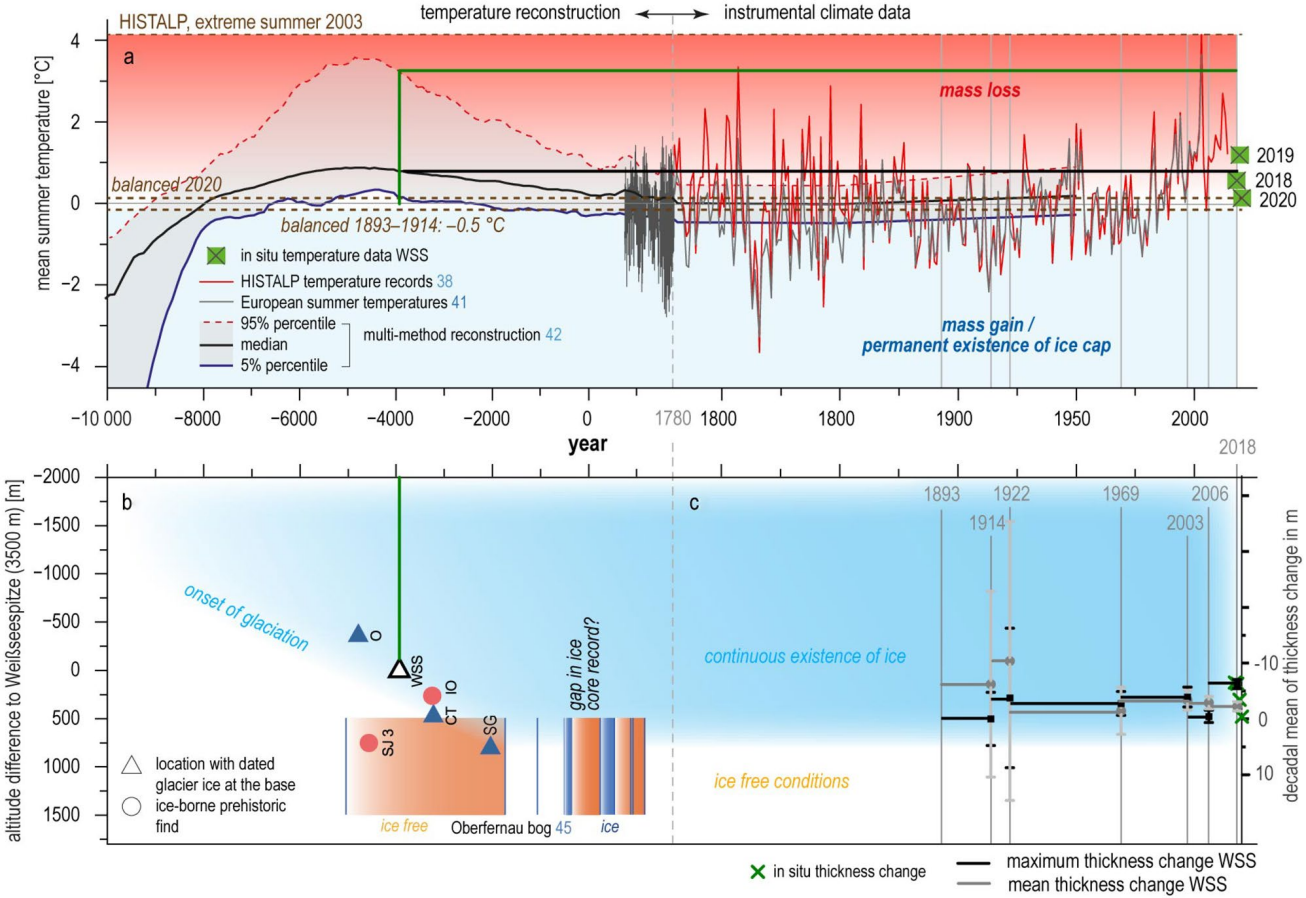
The instrumental summer mean temperatures roughly cover the bandwidth of centennial mean temperatures for the last 10,500 years (**a**), so that measured mass balance (**a**) and thickness changes between 1893 and 2018 (**b**) constrain the conditions needed for the existence of glacier ice and a continuous ice core record at WSS (**c**). Almost balanced conditions, as found in 2020 and between 1893 and 1914, are the minimum requirement for a continuous stratigraphic record, with mass losses of 2017, 2018, 2019, and between 1914 and 2018 resulting in significant ice losses with decadal means of up to 60% of the total length of the ice core in 2017—and therefore must have been rare and/or discontinuous during the length of the record, as today’s accumulation rates can hardly compensate potential ice losses. Therefore, it is not only the onset of neoglaciation which allows us to relate the individual mass loss/balance/gain to the climatic conditions, but also the existence of record gaps in the individual cores at different altitudes. (**b**) Comparing the datings of the onset of glaciation at different elevations (*O* Ortler, *WSS* Weißseespitze, *CT* Chli Titlis, *SG* Schladminger Gletscher) with dated remnants previous to the glaciation (*IO* Iceman Ötzi, *SJ3* Schnidejoch 3) and type localities like the Oberfernau bog allows an altitude-specific sampling of climate events as periods of ice melt and formation once an empirical relation between local mass balance (c, see the data in Table 2) and climate (a) can be established based on a larger ensemble of summit data.

Figure 6a presents the synopsis of Holocene proxy climate records, instrumental and in situ climate data compared to the empirical glaciological evidence on the existence of ice, thickness change and mass balance (Tables 1, 2). For all data samples, increasing length goes along with decreasing resolution. We plotted the mean summer temperatures extrapolated to the altitude of Weißseespitze to compare present to past climate conditions relevant for glacier mass balance. Summer means above 0 °C in 2018, 2019 and 2020 went along with mass losses at the summit, and potentially provide evidence of a statistical correlation of positive temperatures and melt. So do above average thickness loss 2006–2018 and above 0° summer means from 2000 to the end of the record in 2014. The warm summers in the twenty-first century’s instrumental record exceed the dendrochronological temperature series^41^ and the mean, but not the 95% percentile of the multi-method reconstruction^42^. The comparison of the onset of the WSS ice core with the multi-method reconstruction time series confirms the dating of the reformation of ice caps during cooling after the Holocene Optimum. Presuming that a reglaciation of the summit would require positive mass balance rates, which we can now define as conditions cooler than 2020 and similar to 1893–1914 (balanced as shown in Fig. 6c), we could expect local conditions to be cooler than the mean of the multi-method reconstruction. An historical analogue approach like this using sparse empirical evidence to constrain the potential range of local responses to global climate change is often used for complex socioecological systems^43^ as a first step when sufficient data is not available to implement a model output statistics^44^.

The time when cold Alpine ice bodies (Fig. 6b) started to reform follows an altitudinal gradient^15^. This suggests air temperature as a major driver over low regional variabilities in local accumulation^45^. Schladminger glacier formed at the time when the Oberfernau bog in the Stubai Alps^46^ was still ice-free. This was possible, even though Schladminger glacier is located lower, because the amount of precipitation there is higher than in the Stubai Alps^45^. The changes between glacier cover and ice-free in conditions in Oberfernau bog basin points at potential local climate change effects which could be also tackled in Eastern Alpine cores.

At Weißseespitze, the mean annual potential accumulation maximum from 1800 to 2014 is 0.83 m w.e. (if no melt takes place) according to the HISTALP data (mean annual solid precipitation). This is confirmed by the observed maximum snow heights of 2.43 m, assuming an average density of 400 kg/m^3^. At the neighbouring glaciers, snow pits in lower and less wind-exposed locations reveal much higher maximum snow heights ^47^. Of this potential maximum accumulation, 0.59 m w.e. occurs in the same months that we observe increases in snow height today. The HISTALP precipitation signal does not indicate a centennial trend, but annual and decadal variabilities. The in situ effects of the given range of variability are captured by the measurements at Kesselwandferner stake L2, suggesting that multi-annual gaps in the ice core records result not from an absence of accumulation, but from warm summer conditions.

The presence of continuous winter accumulation at WSS is also supported by data from a 20 m deep firn pit on Kesselwandferner at an altitude of 3240 m^48^. It revealed 14 annual layers of firn within the upper 19 m, which were attributed to the accumulation of the years 1963 to 1949, reaching a density of 820 kg/m^3^. In the firn pit, several bands of superimposed ice were found, and water was observed to penetrate and saturate the firn layer. This is not the case at the WSS summit today, as no more firn is present there. So far, no supraglacial runoff system has been observed that would provide geomorphological evidence for melt water flowing on or in the ice body, water storage and penetration is limited to microcracks.

The comparison of the thickness loss at WSS to the age constraint of the firn pit on Kesselwandferner supports an age prior to the atom bomb tests consistent with the lack of tritium in the ice core. The thickness loss between 1969 and 2018 at the summit of Weißseespitze is similar to the depth of the snow pit at Kesselwandferner. Hypothesizing that basically no new layers formed during this period, the loss would therefore expose a surface from about 1950. Taking into account that the summer of 1947 was reported to cause a similar ice loss to 2003, today’s surface likely dates from layers formed prior to 1947. This is supported by the absence of the tritium peak from atmospheric nuclear bomb tests in the 1960s in the ice core^15^. New approaches and dating techniques^49^ may soon allow determining the surface age at ice core sites with present negative mass balance, a problem that has recently become more widely recognized^16,41^.

## Conclusion

The synopsis of in situ mass balance and the related processes, such as wind erosion and ablation, confirms that Weißseespitze summit ice cap is indeed sensitive to climate change. With hardly any temporal variability in accumulation for the last 200 years, the ice archive is very sensitive to warm summer temperatures. Today just a few days of ice melt can trigger negative mass balances with a complete loss of the annual accumulation. The thickness change of the summit ice cap could be constrained to 34.9 ± 10.0 m between 1893 and 2018 by analysing historical maps. This result was confirmed by historical photographs. The present annual melt rates of up to 0.95 m/year measured at single stakes are clearly higher than the average annual mean thickness loss of 0.28 ± 0.08 m/year for 1893 and 2018.

The link between ice core-based mass balance signals and climate is known from using layer thickness as a proxy for past precipitation rates in polar cores^50^. However, in an Alpine summit glacier like Weißseespitze, with a mass balance regime highly sensitive to climate change, this link is far more complex. Reconstructions of Holocene temperatures^42^ and glaciation^51^ point to a fairly pronounced Holocene maximum temperature right before the reglaciation at Weißseespitze started, according to the age constraint of the basal ice layer of (5.9 ± 0.7) ka cal (Fig. 6).

### Future directions and perspectives

The glacio-meteorological settings of Weißseespitze today, in particular the close link between summer temperature and mass loss, finally provide a context that is crucial for further interpretation of mass balance discontinuities. These can be detected in the ice core by visual, chemical and isotopic analysis. Deciphering this unique record of past mass balance will require close chronological control to complement the available constraints from micro-radiocarbon dating^15^.

Just 0.7% of the long-term meteorological stations in mountain regions are located above 3000 m. Few of the high elevation stations are located on summits. The majority of them are located in valleys and influenced by orographic winds which blur climate trends. Because the ice caps to be studied are located on summits and represent free atmosphere temperatures, Alpine ice core research can contribute significantly to the investigation of elevation-dependent warming and related processes^52^. The analysis of 200 years of equilibrium line variability in the European Alps^53^ confirms the potential of the combination of glacier mass balance and HISTALP data for process studies like ours to bridge the gap to Holocene climate and glacier processes.

Next steps in utilizing ice archives for reconstructing the local past climate are consistent sampling at different altitudes, a calibration of the link between instrumental climate records and ice core records by chrono-sequencing annual accumulation history in the age-depth relation and intensified mass balance measurements at the summits, in order to do analogue studies comparing modern to past extremes.

Revealing the pace of past glacier and climate changes is most important in the light of potentially rapid warming during the coming decades. Elevation-dependent warming is assumed to have a great but so far unquantified impact on biodiversity and hydrological regimes, and instrumental data will rarely be available for elevations above 3000 m.

Therefore, the preservation of summit ice and the analysis of mass balance, age sequences and pollen can significantly support adaptation measures like planting climate-resilient protective forest species. This is key for a sustainable future of people living the world’s mountain regions.

## Methods

### In situ mass balance

Point mass balance at Weißseespitze was measured by wooden stakes drilled into the ice^54^ and read several times during the ablation season. In addition to that, snow height was measured by probing and continuously with a sonic ranging sensor at the AWS.

In total, six ablation stakes were distributed over the summit area (Fig. 1b, stakes a–f). Two ablation stakes were installed in 2017 (c, f) at the summit. In 2018, stakes were added at locations a, b, d, e. In 2019, a stake was placed in the hole of the ice coring.

### Measurements of elevation change at the Kesselwandferner summit stake

The metal stakes at KWF were repositioned annually. The initial and the final location of the stake were measured by means of tachymetry prior to 2009 and since then by differential GPS 25. Stake L1 was drilled close to the summit of Fluchthorn at an altitude of 3323.3 m (1968). There, mean annual horizontal flow velocity was 0.6 m/year, with a maximum of 0.7 m. Flow velocities at stake L2 at an altitude of 3296.8 m in 1968 are higher, with an average of 1.7 m/year and a maximum of 2.8 m/year. For both stakes, no local measurements of snow and firn density are available. Both records were the highest stake measurements in the Austrian Alps before the WSS stakes came into operation, and serve only as comparison and validation of the elevation changes recorded in the historical maps.

### Records of meteorological conditions and englacial temperatures at the WSS

An AWS was installed in the summit region at an elevation of 3499 m (location, see Fig. 1) in October 2017. It mainly consists of Campbell Scientific (CS) components and the data logger CR3000. Records of air temperature and humidity (Rotronic-HC2S3), air pressure (CS225), wind speed and direction (Young-05103-45), the energy balance (Huxeflux-NR01), snow accumulation and ice ablation by sonic ranging sensor (CS-SR50a) and ice temperatures in four different depths (CS-225) are taken every minute and stored on 10-min intervals. The snow height refers to the related ice surface at the end of the ablation season as zero. The ice temperature sensors were initially installed in 2017 at the lower end of steam-drilled holes at depths of − 1 m, − 4 m, − 6 m and − 9 m, taking the 2017 ice surface as zero. The two stakes c and f, as well as the stake at the drilling site, are equipped with PT100 temperature probes.

### Positioning

A Tocpon Hiper V DGPS was used to position the ground-penetrating radar (GPR) profiles and stakes. The DGPS raw data, recorded per second, was corrected and adjusted in post-processing with the four closest STPOS (South Tyrolean Reference Station Service) base stations Mals-Malles, Meran-Merano, Bozen-Bolzano and Sterzing-Vipiteno. The achieved vertical and horizontal accuracy of the processed positioning data is within a range of ± 0.5 m. The synchronization of the GPR trace number with the DGPS data was done via the GPS time under consideration of the current 18 leap seconds to the GSSI-recorded UTC time.

### GPR and bedrock topography

The ice thickness at the Weißseespitze summit was measured with GPR on 8 June 2017. Because of the expected low ice thicknesses, a GSSI 3102A antenna with a central frequency of 500 MHz was used. The radar sections were recorded at the uppermost area of the summit along several cross profiles (Fig. 1, Supplementary Fig. S4) at an interval of 4 scans per second and 1024 samples per scan. The GPR signal was processed with ReflexW software (see the radargrams in Fig. S5) and a time-to-depth conversion with a mean signal velocity of 0.168 m/ns, arriving at an uncertainty of 10% of the ice thickness^55^.

The bedrock topography of the summit was calculated from the GPR measurements and a bilinear interpolation between the bedrock interpretation of the GPR profiles, the glacier outlines as zero ice thickness and taking into account the surrounding ice-free topography from the 2018 DEM^55^. The bedrock elevation is consistent with the ice core drillings. The vertical accuracy of the DGPS-measured surface elevation is ± 0.5 m, so that the accuracy of the bedrock elevation is constrained by surface elevation uncertainty and the ice thickness uncertainty of ± 10%.

### Gridded surface elevation and thickness change data

Basic data for the calculation of past elevation and volume changes are provided by different available DEMs as given in Table 1. DEMS prior to 2006, with a pixel size of 5 × 5 m, are based on aerial photogrammetry and show vertical uncertainties better than ± 1.9 m^56^. From 2006 onwards, the DEMs are based on high-resolution LiDAR data with 1 × 1 m pixel size. For the DEM of 2006, the uncertainty is ± 0.1 m^56^. The standard deviation at control areas for the DEM of 2018 was 0.032 m^57^. The effects of a seasonal snow cover, to be added to the nominal accuracy of the DEMs, can be estimated by the maximum snow cover recorded at the AWS to be about 2 m.

The DoDs (DEM of Difference) were calculated in ArcMap with the “minus” tool and took into account the basic resolution of the data and exact overlapping raster cells. The volume change of the summit ice cap was calculated for the area where the bedrock is above the 3470 m.a.s.l. contour line by summing up the volume changes in each pixel of the DoD grids.

### Historical maps, photographs and climate data

For the time before 1969, elevation and shape of the ice cap at Weißseespitze are recorded in historical maps and a number of photographs. Although this summit has been more remote and less prominent than others (for example, nearby Weißkugel summit), the Gepatschferner glacier has been subject to early glaciological surveys. The summit of Weißseespitze was also surveyed during federal mapping campaigns and is named and indicated in maps from the third survey of Tyrol onwards, which took place during 1870–1887 and quotes elevation for the rock outcrops and two edges leading down from the summit. Although early measurements of elevation have been challenging and generally have uncertainties of several metres, the data fits remarkably well to the historical photographs and today’s high-resolution data at rock outcrops. Reprocessing the historical map of Hochjochferner based on the original photogrammetric records returned a standard error in elevation of the primary observatory network (triangulation points) of ± 0.21 m^58^, an uncertainty of ± 1–2 m at the glacier tongue, where tacheometry was applied, and an uncertainty of ± 0.10 m at photogrammetrically processed upper parts of the glacier. For our analysis, we used maps where tacheometric surveys are indicated by cartographic symbols.

We compared present meteorological conditions at the summit ice cap to homogenized HISTALP climate data^40^ and the Jungfraujoch temperature records^59^ (Table S5). The gridded monthly mean HISTALP data sets are available in 1° and 5ʹ spatial resolution and are based on 557 instrumental climate records dating back to 1760 AD. The 1° HISTALP grid point next to Weißseespitze is located at 46.83 N, 10.75 E, at an elevation of 3160 m, for the location of the 5ʹ resolution grid points NE, NW, SE, and SW, see Table S6. Temperatures at all HISTALP grid points are higher than at the AWS, which is located at 3499 m, so that the temperatures had to be extrapolated to the summit altitude.

We extrapolated the HISTALP data to the AWS altitude in two ways, by adding the long-term difference to Jungfraujoch records and by using the lapse rate derived from the HISTALP points at different altitudes^60^. The respective lapse rates are listed in Table S6.

For the long-term station at Jungfraujoch in Switzerland (3571 m, 46° 32.9ʹ N, 7° 59.1ʹ E), homogenized monthly mean temperatures are available from 1933 onwards. During summer months June, July and August in the overlapping period 1933 to 2014, the difference between Jungfraujoch and Weißseespitze temperature means is 2.3 °C, with a standard deviation of 0.2 °C. This difference was added to the HISTALP monthly mean for a first rough estimate of melting conditions at the summit during the HISTALP period. For the summer means (June, July, August), when most of the melt events occur, the Pearson correlation coefficient between Jungfraujoch and the extrapolated HISTALP grid point is 0.94, for annual means 0.97. As the resulting extrapolated records are very similar (Table S7, Figs. S2, S3), we can very confidently describe local climatology by the adapted HISTALP series.

Potential snow accumulation was calculated for the HISTALP grid point from the fraction of solid precipitation and total precipitation, assuming that precipitation in December, January and February was eroded by winds as is the case today. No correction to the altitude was applied to the amount of precipitation or the fraction of solid precipitation.

## Supplementary Information

Supplementary Information.

## Data Availability

The DEMs of 2006 and 2018 have been published by the Federal Administration of Tyrol and available via the open government portal data.tirol.gv.at. All other data are available via pangaea.de ( doi:10.1594/PANGAEA.939817 and doi:10.1594/PANGAEA.939830 ).
