# Supplementary material for: Contemporary mass balance on a cold Eastern Alpine ice cap as a potential link to the Holocene climate
Source: Sci Rep. 2022 Jan 25;12:1331. doi: 10.1038/s41598-021-04699-2 (PMC8789798; doi:10.1038/s41598-021-04699-2)
Supplement: Supplementary file 1 — Supplementary Information. [file 41598_2021_4699_MOESM1_ESM.docx]

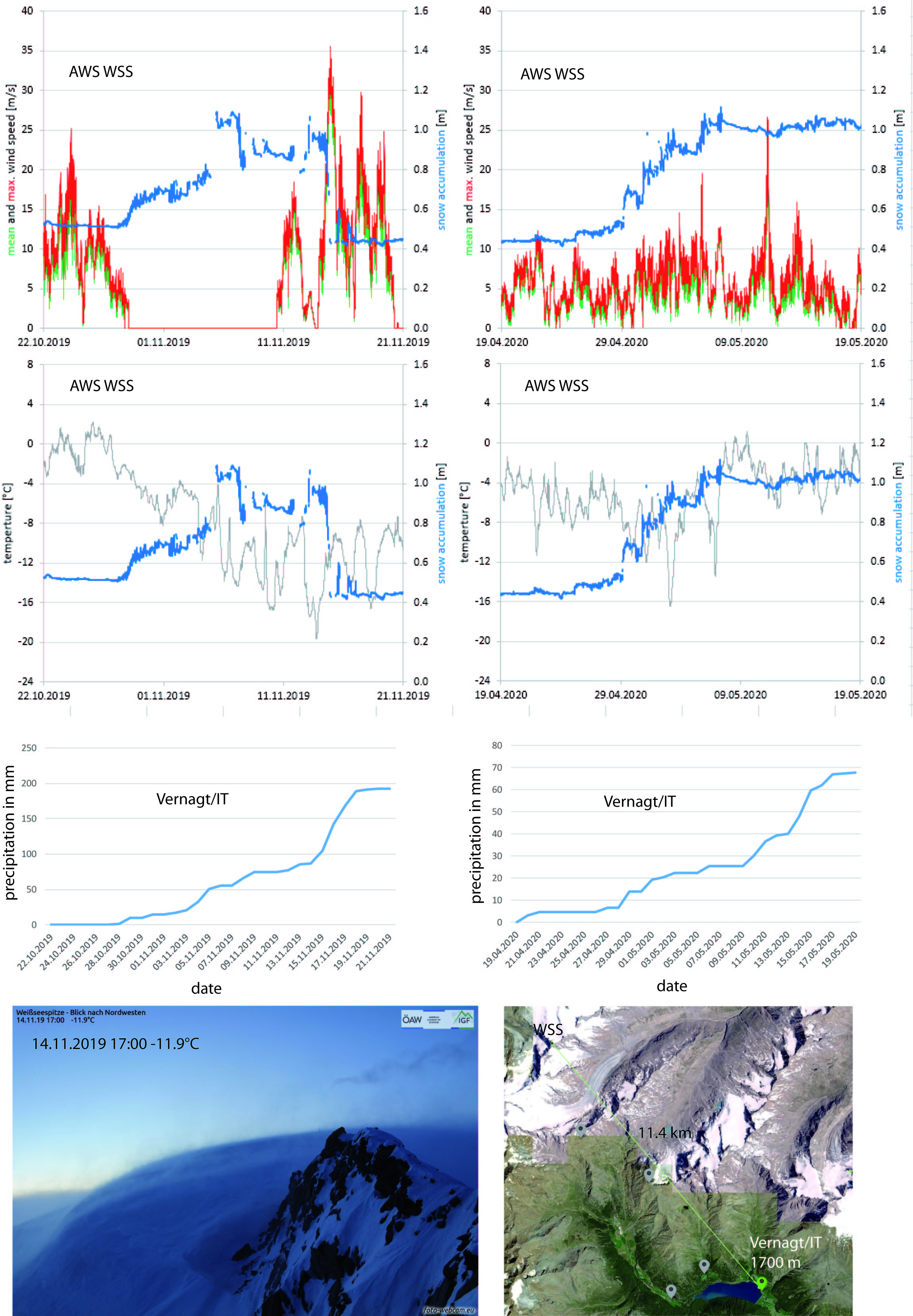


Figure S1: Wind erosion removes snow at Weißseespitze summit despite ongoing precipitation recorded at the nearby weather station of Vernagt (Italy), provided by the open government data portal of the autonomous province of Bozen/Bolzano <https://wetter.provinz.bz.it/download-messdaten.asp>.

Table S1: Minimum, maximum and mean ice temperatures (T_ice_) at depths of 9, 6 and 1 m (with the ice surface in 2017 defined as zero), as well as air temperature (T_air_), measured at the AWS for the glaciological years Nov. 2017-Oct. 2018 (2018) and Nov. 2018-Oct. 2019 (2019 and 2020. *The positive maximum temperature in 2019 at -1m was caused by melting along the instrument cable and air temperature. **Maximum snow height until June 2020.

|  | T_ice_ (-9m) in °C | | | T_ice_ (-6m) in °C | | | T_ice_ (-1m) in °C | | | T_air_ in °C | | |
| --- | --- | --- | --- | --- | --- | --- | --- | --- | --- | --- | --- | --- |
|  | min | max | mean | min | max | mean | min | max | mean | min | max | mean |
| 2018 | -3.4 | -2.8 | -3.1 | -4.9 | -2.2 | -3.8 | -6.0 | 0.0 | -3.9 | -33.1 | 9.5 | -7.6 |
| 2019 | -3.6 | -2.6 | -3.2 | -5.4 | -2.3 | -4.3 | -6.9 | *0.2** | -4.5 | -26.4 | 11.1 | -6.1 |
| 2020 | -3.7 | -2.8 | -3.3 | -6.0 | -2.3 | -4.1 | -9.0 | *0.3** | -4.5 | -24.0 | 11.8 | -6.1 |

Table S2: Maximum elevation (H_max_) of the Weißseespitze summit from DEMs and historical maps, as well as the mean ice thickness ($\bar{d}$) and ice volume (V) for the specific years until 1969 above the 3470 m bedrock contour line.

| DEM year | H_max_ [m a.s.l] | | ± | raster [m] | $\bar{d}$ [m] | V [10^6^ m^3^] | Map source |
| --- | --- | --- | --- | --- | --- | --- | --- |
| 2018 | 3499.1 | | 0.03 | 1x1 | 12.1 | 0.384 | 52 |
| 2006 | 3506.6 | | 0.1 | 1x1 | 14.8 | 0.469 | 37 |
| 1997 | 3507.0 | | 1.9 | 5x5 | 18.3 | 0.579 | 37 |
| 1969 | 3518.0 | | 1.9 | 5x5 | 27.7 | 0.878 | 37 |
|  | | Elevation from historical maps | | | | |  |
| year | H_max_ [m a.s.l] | | ± | $\bar{d}$ [m] | | |  |
| 1971 | 3517 | | 1.9 |  | | | 34 |
| 1922 | 3531 | | 10 | 35±20 | | | 27 |
| 1914 | 3534 | | 10 | 43±20 | | | 36 |
| 1893 | 3534 | | 10 | 30±25 | | | 35 |

Table S3: Total and annual elevation (h) and volume (V) changes of DoDs (DEM of Difference) for the area above the 3470 m bedrock contour line.

| DoD years | | Raster size [m] | Δh [m] | ΔV [m³] | Δh/a [ma^-1^] | ΔV/a [m^3^a^-1^] |
| --- | --- | --- | --- | --- | --- | --- |
| 2018 | 2006 | 1x1 | -2.7 | -85828 | -0.23 | -7152 |
| 2018 | 1997 | 5x5 | -6.1 | -193469 | -0.29 | -9213 |
| 1997 | 1969 | 5x5 | -9.4 | -297755 | -0.34 | -10634 |

Table S4: Monthly mean temperature as recorded at the AWS on the WSS summit.

|  | Monthly mean temperature in °C | | | | | | | | | | | |
| --- | --- | --- | --- | --- | --- | --- | --- | --- | --- | --- | --- | --- |
| Year | 1 | 2 | 3 | 4 | 5 | 6 | 7 | 8 | 9 | 10 | 11 | 12 |
| 2017 |  |  |  |  |  |  |  |  |  |  | -10.6 | -14.8 |
| 2018 | -12.2 | -17.7 | -12.9 | -6.2 | -3.5 | -1.3 | 1.1 | 1.9 | -0.4 | -4.2 | -5.8 | -11.2 |
| 2019 | -17.8 | -10.3 | -11.8 | -9.1 | -7.8 | 0.9 | 1.3 | 1.4 |  | -3.3 | -10.5 | -10.8 |
| 2020 | -9.8 | -11.5 | -12.0 | -6.6 | -5.3 | -1.6 | 0.6 | 1.4 | -1.0 | -6.9 | -5.5 | -12.3 |
| 2021 | -16.6 |  |  |  |  |  |  |  |  |  |  |  |

Table S5: The HISTALP record of solid precipitation and monthly mean temperatures at the SE grid point closest to the Weißseespitze, and the record adapted to the Jungfraujoch record. WSS adapted…temperature extrapolated to 3499 m, T mean … monthly mean temperature.

Table S6: Location, altitude (z), altitude difference (dz) of the HISTALP gridpoints NE, NW, SE and SW of the 5’ resolution grid to the HISTALP grid point (HISTALP) of the 1° resolution grid, the automatic weather station (AWS) and Jungfraujoch (JFJ). For all records, the elevation difference to the summit of Weißseespitze (dz _WSS_ ), the temperature gradient dT calculated respective to 5’ gridpoint SE, and the average annual (T_annual_) and summer (T_summer_) temperatures, with summer defined as the months of June, July and August are given.

|  | r | E | N | Z | dz | dz _WSS_ | dT | | | T_annual_ | T_summer_ |
| --- | --- | --- | --- | --- | --- | --- | --- | --- | --- | --- | --- |
|  |  | ° | ° | m | m | m | °C | | | °C | °C |
| HISTALP | 1° | 10.7500 | 46.8300 | 3160 |  |  |  |  |  | -5.7 | 1.1 |
| NE | 5' | 10.7500 | 46.9167 | 2236 | 924 | 1263 | -0.0058 | ± | 0.0017 | -7.8 | -1.2 |
| NW | 5' | 10.6667 | 46.9167 | 3036 | 124 | 463 | -0.0053 | ± | 0.0016 | -7.6 | -0.9 |
| SE | 5' | 10.7500 | 46.8333 | 3160 | 0 | 339 |  |  |  | -7.9 | -1.3 |
| SW | 5' | 10.6667 | 46.8333 | 2380 | 780 | 1119 | -0.0060 | ± | 0.0017 | -8.0 | -1.2 |
| AWS |  | 10.7180 | 46.8463 | 3499 |  |  |  |  |  |  |  |
| JFJ |  | 7.9850 | 46.5483 | 3571 |  | 72 | -0.0319 | ± | 0.0870 | -7.5 | -1.2 |

Table S7: The annual T_annual_ and summer temperatures T_summer_ of the extrapolated HISTALP 5’ grid points, averaged over the full length of records (1770-2014), are very similar for all methods applied (HISTALP gridpoints NE, NW, SW: gradient approach, Jungfraujoch record JFJ: temperature difference to HISTALP added.)

| Grid point | T_annual_ | T_summer_ |
| --- | --- | --- |
|  | °C | °C |
| NE | -7.8 | -1.2 |
| NW | -7.6 | -0.9 |
| SW | -7.9 | -1.3 |
| JFJ | -8.0 | -1.2 |

Figure S2: Time series of annual mean temperature, extrapolated with different methods.

Figure S3: Time series of summer mean temperature, extrapolated with different methods.


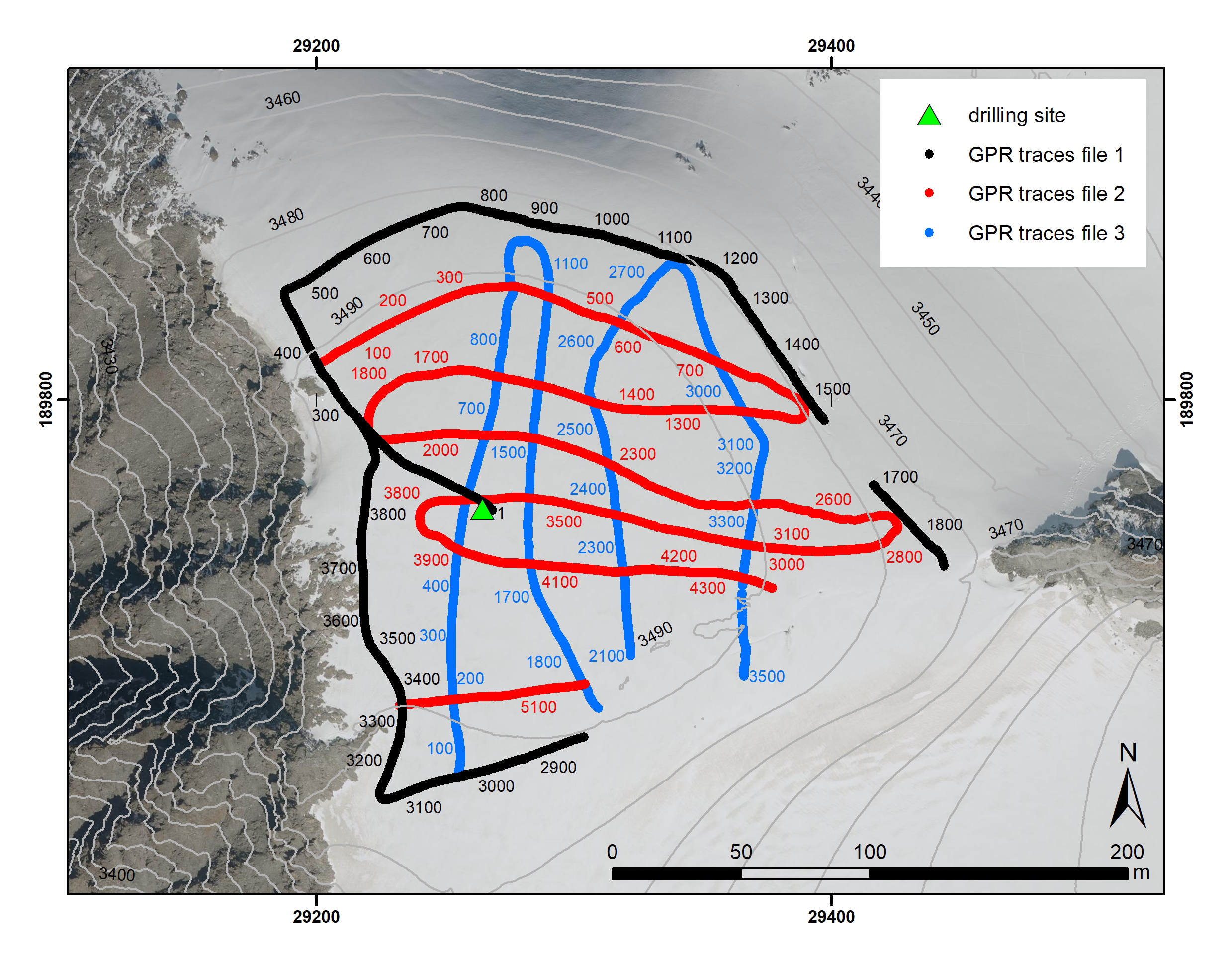


Figure S4: Location of GPR profiles on WSS summit. Map generated with ArcMap 10.6.1. (https://www.esri.com/en-us/arcgis/products/arcgis-desktop/overview).


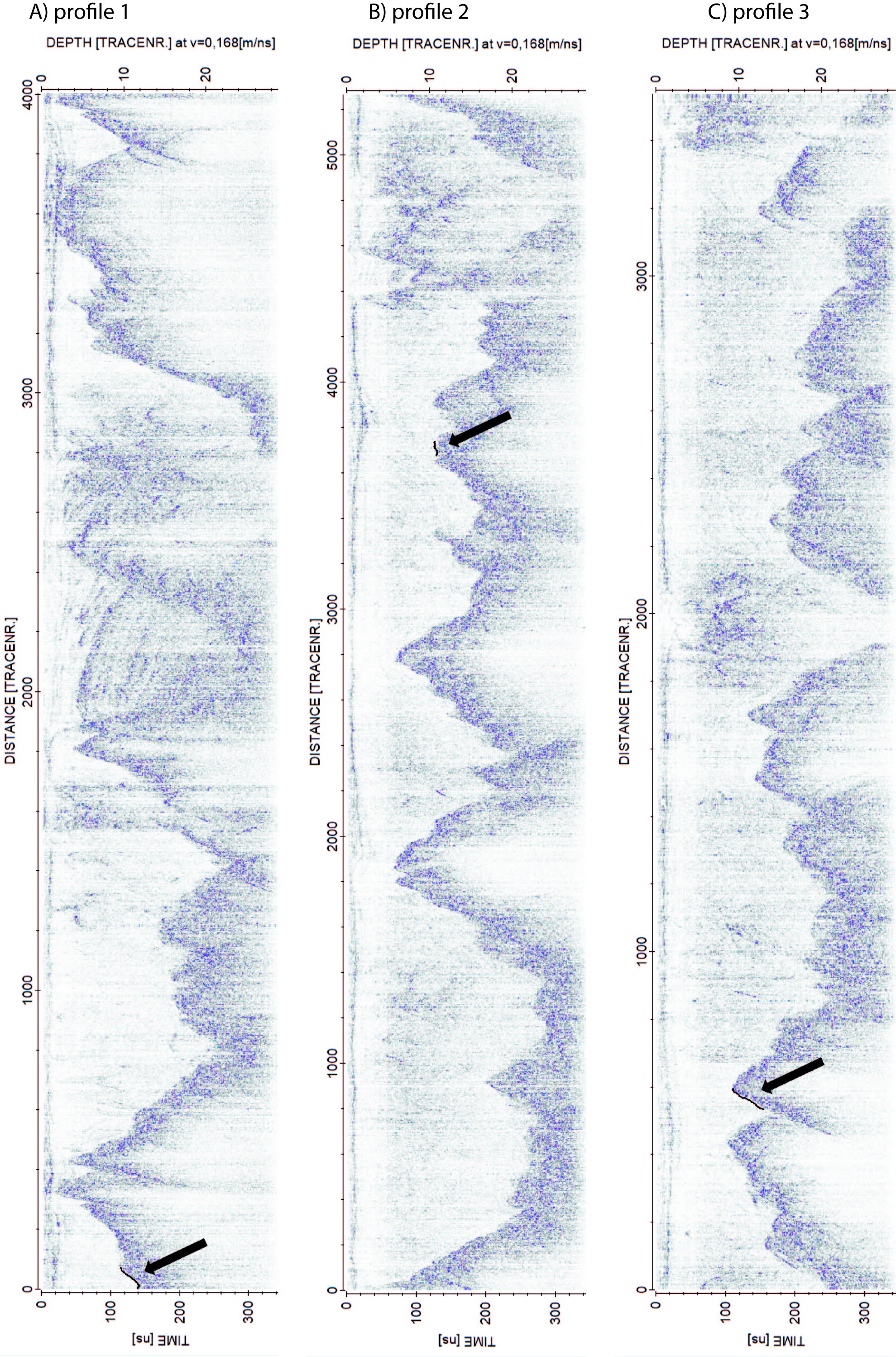


Figure S5: Radargrams of the GPR profiles 1-3 at WSS with the arrow indicating the trace numbers next to the drilling location.

Long-term measurements of the altitudinal gradient of precipitation in the inner Ötztal would be available, as well as long-term measurements of accumulation in snow pits of the Hintereisferner and Vernagtferner glaciers 1. The accumulation measured in the snow pits at about 3350 m altitude on Hintereisferner is about 1.5 times the precipitation recorded in the rain gauge at 3026 m. The respective snow heights between 4.5 and more than 6 m are much higher than for the WSS summit at 3500 m. Precipitation thus clearly increases with altitude within the operational range of rain gauges. This is not true for accumulation, which decreases again with altitude after reaching a topographically induced maximum at the glacier base as evident from snow probings during the annual mass balance surveys 2.

1 Strasser, U., Marke, T., Braun, L., Escher-Vetter, H., Juen, I., Kuhn, M., Maussion, F., Mayer, C., Nicholson, L., Niedertscheider, K., Sailer, R., Stötter, J., Weber, M., and Kaser, G.: The Rofental: a high Alpine research basin (1890–3770 m a.s.l.) in the Ötztal Alps (Austria) with over 150 years of hydrometeorological and glaciological observations, Earth Syst. Sci. Data, 10, 151–171, https://doi.org/10.5194/essd-10-151-2018, 2018.

2 [Fischer, Andrea](https://doi.pangaea.de/10.1594/PANGAEA.818898)**; Markl, Gerhard;** [Kuhn, Michael](https://doi.pangaea.de/10.1594/PANGAEA.818898) **(2013):** Glacier mass balances and elevation zones of Hintereisferner, Ötztal Alps, Austria, 1952/1953 to 2010/2011. [*Institut für Interdisziplinäre Gebirgsforschung der Österreichischen Akademie der Wissenschaften, Innsbruck*](https://ror.org/009syct46), PANGAEA, <https://doi.org/10.1594/PANGAEA.818898>
